# Long-term mycophenolate monotherapy in human leukocyte antigen (HLA)-identical living-donor kidney transplantation

**DOI:** 10.1186/2047-1440-3-4

**Published:** 2014-02-03

**Authors:** Blanca Gascó, Ignacio Revuelta, Ana Sánchez-Escuredo, Miquel Blasco, Federico Cofán, Nuria Esforzado, Luis F Quintana, María José Ricart, José Vicente Torregrosa, Josep M Campistol, Federico Oppenheimer, Fritz Diekmann

**Affiliations:** 1 Servicio de Nefrología y Trasplante Renal, Hospital Clínic, Villarroel 170 08036 Barcelona, Spain; 2 Servicio de Nefrología, Hospital Universitario Virgen Macarena, Avd. Dr. Fedriani, 3, 41007 Sevilla, Spain

## Abstract

**Methods:**

We analyzed all PRA-negative patients who received a first kidney transplant from an HLA-identical living donor. The patients received no antibody induction. An intraoperative bolus of 500 mg of methylprednisolone was administered. Then, steroid therapy was withdrawn within one week. Tacrolimus and mycophenolate treatment were started 3 days before transplantation with tacrolimus target levels of 4 to 8 ng/mL. In the absence of rejection, tacrolimus was withdrawn between 3 and 12 months post-transplant to reach mycophenolate mofetil monotherapy of 2 g/day or equivalent.

**Results:**

Six patients were treated with the above protocol. At last follow-up, graft and patient survival were 100%. MDRD glomerular filtration rates were 54, 60, and 62 mL/min at 3 months, 12 months and last follow-up, respectively. None of the patients developed PRA post-transplant. One episode of acute rejection Banff IA occurred 9 years after transplantation due to non-adherence with good outcome after treatment. The mean number of concomitant drugs given with mycophenolate was 2.6. Four patients needed antihypertensive drugs.

**Conclusion:**

Steroid-free *de novo* treatment and calcineurin-inhibitor weaning with mycophenolate monotherapy is feasible in first HLA-identical kidney transplantation from a living sibling.

Although recipients of a first HLA-identical living-donor kidney transplant seem to need less immunosuppression, there are no guideline recommendations for these patients, and few prospective trials are available.

## Introduction

Only a very small group of patients receive kidney transplants from an HLA-identical sibling. However, these patients show excellent long-term graft outcome and seem to need significantly less immunosuppression compared with recipients from nonHLA-identical donor kidneys [1-5]. In general, withdrawal or minimization of immunosuppressive treatment has been one of the objectives in low immunological risk patients [6,7]. However, since only a minority of kidney transplants is performed among HLA-identical siblings, few studies have been carried out in these patients, and ‘how much is less’ remains poorly defined. No clear recommendation is given in the KDIGO guidelines for immunosuppression in kidney transplantation from a living HLA-identical donor [8].

The aim of this report is to increase the data available on HLA identical kidney transplantation in order to avoid unnecessary and potentially dangerous over-immunosuppression in these patients.

We describe our experience of 10 years of HLA-identical living donor kidney transplantation without induction treatment, a 7-day course of steroid treatment, calcineurin inhibitor withdrawal during the first year and mycophenolate monotherapy as maintenance treatment.

### Patients and methods

We report retrospectively on all consecutive PRA-negative patients from our center who received a first kidney transplant from an HLA-identical living donor between January 2003 and December 2010. This retrospective analysis was approve by the Review Board of Clinical Investigation Hospital Clínic, and the patients gave informed consent. Inclusion criteria were HLA identity, living donor transplant, first transplant, and absence of HLA antibodies as tested with Luminex™ HLA antibody test class I and II (OneLambda, Canoga Park, California, USA). Exclusion criteria were repeat transplant, double transplant, and Luminex™ class I or II positivity. The patients received no antibody induction and an intraoperative methylprednisolone bolus of 500 mg. On postoperative day 1 (POD1), they received 125 mg of methylprednisolone intravenously (i.v.), and on POD2 the patients were given 0.5 mg/kg of methylprednisolone. Then, the patients continued with oral prednisone until abrupt withdrawal after POD6. Tacrolimus (target levels of 4 to 8 ng/mL) and mycophenolate treatment (500 mg twice per day (BID) pre-transplant and 1 g BID post-transplant or equivalent) were started 3 days before transplantation. Post-transplant tacrolimus concentrations were kept between 4 and 8 ng/mL. In the year 2009 our unit started a protocol biopsy program with biopsies at 3 and at 12 months for all kidney transplant recipients, including patients who received a transplant from an HLA-identical sibling from 2009 onwards. In the absence of clinical (and histopathological) signs of rejection, tacrolimus was slowly withdrawn between 3 and 12 months post-transplant to reach mycophenolate monotherapy of 1 g BID of CellCept™ or 720 mg BID of Myfortic™.

Valganciclovir was given as CMV prophylaxis in all donor positive/recipient negative patients until POD 90. Otherwise, pre-emptive therapy was performed. All patients received sulfamethoxazole/trimethoprim prophylaxis for 6 months.

Routine follow-up was performed with weekly visits in the outpatient department until POD30, then every 2 weeks until POD90 and monthly visits thereafter until 1 year. After 1 year, follow-up was performed every 3 months. Graft function was evaluated according to the Modification of Diet in Renal Disease equation (MDRD). Body weight and blood pressure were recorded, and routine blood tests were performed according to our center protocol.

### Statistical analysis

Values are described as mean, standard deviation and range. The Wilcoxon test was performed in order to detect statistically significant differences. All *P* values were obtained as two sided and were considered to be significant if <0.05.

## Results

Six PRA-negative first kidney transplant recipients who received a kidney from an HLA-identical living donor between 1 January 2003 and 31 December 2010 were identified (Table 1). They had a mean follow-up of 73.3 ± 36 months (33 to 121). Mean recipient age at transplantation was 49 ± 3 years; mean donor age was 48.7 ± 3 years. Causes of end-stage renal disease included IgA nephropathy (n = 2), hypertensive nephropathy (n = 3), and vesicoureteral reflux (n = 1). All but one patient were transplanted pre-emptively. One patient had been on hemodialysis for 54 months prior to transplantation. No patient showed pre-transplant anti-HLA antibodies, and no HLA antibodies were detected after transplantation by Luminex technology.

**Table 1:** Patient characteristics and clinical outcome

	**Recipient 1**	**Recipient 2**	**Recipient 3**	**Recipient 4**	**Recipient 5**	**Recipient 6**
**Recipient age (yr)**	40	33	62	64	50	45
**Recipient sex**	Female	Male	Male	Male	Male	Male
**Initial disease**	Hypertensive nephropathy	IgA Nephropathy	IgA Nephropathy	Hypertensive nephropathy	Hypertensive nephropathy	Vesicoureteral reflux + left kidney atrophy
**Dialysis (months)**	0	0	54	0	0	0
**Donor age (yr)**	44	39	61	50	49	49
**PRA**	0	0	0	0	0	0
**Renal biopsy 3 mo**	Not performed	Not performed	Not performed	Not performed	Normal	Normal
**SCr at 3 mo (mg/dl)**	1.4 mg/dL	1.5 mg/dL	1.7 mg/dL	1.2 mg/dL	1.6 mg/dL	1.3 mg/dL
**eGFR (mL/min/1.73 m2)**	44	57	44	65	49	63
**Biopsy at 1 year**	Not performed	Not performed	Not performed	Not performed	Normal	Normal
**SCr at 1 yr**	1.2 mg/dl	1.4 mg/dl	1.6 mg/dl	1.2 mg/dl	1.3 mg/dl	1.2 mg/dl
**eGFR (mL/min/1.73 m2)**	53	62	47	65	62	69
**Follow-up (mo)**	121	98	96	54	38	33
**Renal function at last follow-up:**						
**SCr at last follow-up (mg/dL)**	1.3 mg/dl	1.3 mg/dl	1.2 mg/dl	1.1 mg/dl	1.4 mg/dl	1.3 mg/dl
**eGFR (mL/min/1.73 m2)**	47	66	61	71	58	66
**Treatment at last follow-up**	Mycophenolate mofetil 750 mg/12 h + Irbesartan 300 mg/24 h + Saccharate iron oxide 40 mg/24 h.	Mycophenolate sodium 720 mg/12 h + Bisoprolol 10 mg/24 h + Ranitidine 300 mg/12 h.	Mycophenolate mofetil 1000 mg/12 h + Irbesartan 300 mg/24 h + Levothyroxine 50 mcg/24 h + Atorvastatin 20 mg/24 h + Pantoprazole 40 mg/24 h.	Mycophenolate mofetil 1000 mg/12 h + Telmisartan 40 mg/24 h + Repaglidina 0.5 mg/12 h + Acenocoumarol + Pantoprazole 40 mg/24 h + Benzbromarone 100 mg/24 h.	Mycophenolate sodium 720 mg/12 h + Acetylsalicylic acid 100 mg/24 h + Pantoprazole 20 mg/24 h + Bromazepam 3 mg/24 h.	Mycophenolate mofetil 1000 mg/12 h

IgA: Immunglobulin A; mg: milligram; h: hour; dl: deciliter.

Patient and graft survival was 100% at 1 year and at last follow-up.

Mean serum creatinine was 1.45 ± 0.19 mg/dL at 3 months, 1.32 ± 0.16 mg/dL at 1 year, and 1.26 ± 0.10 mg/dL at last follow-up corresponding to a glomerular filtration rate (MDRD) of 54 ± 9.3, 60 ± 8.1 and 62 ± 8.4 mL/min, respectively. The difference in kidney function between 3 months and last follow-up was statistically significant (*P* = 0.042 for creatinine and 0.043 for MDRD). Median proteinuria was 194 mg/day (Quartile 1 (Q1): 129; Quartile 3 (Q3): 268; interquartile range (IQR): 139) at 3 months and 280 mg/day (Q1: 155; Q3: 397; IQR: 242) at 1 year. Mean blood pressure at 1 year was 131/80 mmHg. Four patients were on antihypertensive drugs at last follow-up.

One patient with unknown native kidney disease developed late acute rejection Banff IA at 9 years post-transplantation after taking mycophenolate on an irregular basis during the preceding months. This patient showed an increase of serum creatinine from 1.2 mg/dL to 1.4 mg/dL at the time of rejection and an increase of proteinuria and was treated with a bolus of 250 mg methylprednisolone each on three consecutive days and subsequent steroid tapering during the following 2 weeks and returned to his baseline graft function and proteinuria at last follow-up. No antibodies were detected in the Luminex™ screening at time of rejection or afterwards.

No unplanned rehospitalizations were necessary after transplantation in any of the patients. No major abnormalities of the hemogram or lipid profiles occurred.

One patient had a history of pre-transplant diabetes and remained on oral antidiabetics after transplantation.

## Discussion

We observed excellent outcomes in first HLA-identical living-donor kidney transplant recipients on mycophenolate monotherapy.

Living donor kidney transplantation from HLA-identical siblings only accounts for approximately five percent of all living-donor renal transplants in the United States. The percentage is even lower taking into account all renal transplants performed including transplants from cadaveric donors [9]. On the other hand transplants from HLA-identical siblings achieve excellent long-term outcomes compared with HLA-mismatched living-donor renal transplants [10,11]. According to the UNOS database these transplants achieve 5-year graft and patient survival rates of 87.5% and 95%, respectively [12]. One of the possible reasons for this might be the fact that acute rejection rates in transplantation from HLA-identical siblings are significantly lower than in transplant recipients from nonHLA-identical donors [13]. However, as can be seen from one of our patients, a minimal amount of immunosuppression still remains necessary even years after HLA-identical transplantation. This patient developed mild cellular rejection due to non-adherence.

In most centers, more than 85% of kidney graft recipients receive a long-term therapy containing a calcineurin inhibitor [14]. On the other hand long-term calcineurin inhibitor therapy is associated with severe side effects such as cardiovascular disease and cancer. Since only a small percentage of recipients of living-donor kidneys receive transplants from HLA-identical siblings there are no systematic randomized controlled trials available.

Walker and colleagues published a study on 20 HLA-identical living-donor kidney transplants who were treated without steroids and with low-dose calcineurin-inhibitors and the mTOR inhibitor, rapamycin. Tacrolimus was withdrawn at month 3 and SRL at 1 year, and the patients were left on mycophenolate monotherapy after 1 year, which was considered to be safe with a follow-up period of 18 months [15]. Venot and colleagues report on seven HLA-identical living-donor transplant recipients who received polyclonal antibody induction and mycophenolate (six cases) or rapamycin (one case) monotherapy, and achieved excellent results [16]. Leventhal and colleagues recently published the results of a tolerance induction trial in transplant recipients from HLA identical siblings. The patients were treated with alemtuzumab, donor hematopoietic stem cells, tacrolimus and mycophenolate, and later were converted to sirolimus. Finally, complete drug withdrawal was performed at 24 months. Five out of ten patients with at least 36 months of follow-up had the immunosuppression successfully withdrawn, two had disease recurrence and three subclinical rejections [17]. Van der Wetering and colleagues chose 29 out of 83 kidney transplant recipients from an HLA-identical donor and converted them from a dual therapy consisting of steroids in combination with either azathioprine or mycophenolate to steroid monotherapy [18]. One patient developed JC-virus nephropathy with subsequent graft loss, and one patient refused further reduction of immunosuppression. In their cohort four patients on steroid monotherapy developed recurrence of the original disease, however, only one of these patients showed a transient decline of graft function. The observation period was 24 months. Bartucci and colleagues converted 11 HLA-identical kidney transplant recipients successfully from CNI-based treatment to azathioprine monotherapy without deterioration of kidney function in the observation period of 76 months; however, they state that the possible benefits must be carefully balanced against an associated risk of acute rejection [19].

Our data contributes to those reported in the literature and might help define a potential group of recipients of kidney grafts from HLA-identical siblings, who could be on long-term mycophenolate monotherapy, that is, recipients of a first kidney transplant without anti-HLA antibodies (negative PRA testing by cytotoxicity and negative Luminex™ testing).

## Conclusion

A stepwise reduction of immunosuppression to reach mycophenolate monotherapy might be a practical and reasonably safe method that could offer a sufficient level of immunosuppression to those patients receiving a first HLA-identical kidney transplantation from a living sibling without unnecessary exposure to expensive and potentially harmful treatments.

## Abbreviations

BID: twice per day; HLA: human leucocyte antigen; i.v.: intravenous; MDRD: Modification of Diet in Renal Disease; POD1: postoperative day 1.

## Competing interests

The authors declare that they have no competing interests.

## Authors’ contributions

BG collected and reviewed the data, performed the analysis and contributed to writing the manuscript. IR, AS, MB, FC, NE, LQ, MR, JT collected data, contributed to data analysis and critically reviewed the manuscript. JC and FO designed the treatment, collected data and critically reviewed the manuscript. FD designed the study, collected data, performed the data analysis and wrote the manuscript. All authors read and approved the final manuscript.
